# Factors influencing health professionals' use of high-flow nasal cannula therapy for infants with bronchiolitis – A qualitative study

**DOI:** 10.3389/fped.2023.1098577

**Published:** 2023-03-16

**Authors:** Sharon L. O’Brien, Libby Haskell, Emma J. Tavender, Sally Wilson, Meredith L. Borland, Ed Oakley, Stuart R. Dalziel, Fenella J. Gill

**Affiliations:** ^1^ Emergency Department, Perth Children's Hospital, Nedlands, WA, Australia; ^2^ School of Nursing, Faculty of Health Sciences, Curtin University, Bentley, WA, Australia; ^3^ Children’s Emergency Department, Starship Children’s Hospital, Auckland, New Zealand; ^4^ Department of Paediatrics: Child and Youth Health, University of Auckland, Auckland, New Zealand; ^5^ Clinical Sciences Theme, Murdoch Children’s Research Institute, Parkville, VIC, Australia; ^6^ Departments of Paediatrics and Critical Care, University of Melbourne, Parkville, VIC, Australia; ^7^ Divisions of Emergency Medicine and Paediatrics, School of Medicine, University of Western Australia, Crawley, WA, Australia; ^8^ Emergency Department, Royal Children’s Hospital, Parkville, VIC, Australia; ^9^ Departments of Paediatrics and Critical Care, University of Melbourne, Parkville, VIC, Australia; ^10^ enAble Institute, Curtin University, Bentley, WA, Australia; ^11^ Nursing Research, Perth Children’s Hospital, Child & Adolescent Health Service, Nedlands, WA, Australia

**Keywords:** implementation intervention, high-flow nasal cannula therapy, theoretical domains framework, bronchiolitis, nurses, doctors, evidence-based care, paediatric

## Abstract

**Aim:**

To explore the factors influencing the use of high-flow nasal cannula (HFNC) therapy for infants with bronchiolitis.

**Design:**

Qualitative approach using semi-structured interviews.

**Methods:**

The semi-structured interviews (face-to-face or virtual) were conducted between September 2020 and February 2021. Deductive content analysis was used to map key influencing factors for use of HFNC therapy to the Theoretical Domains Framework (TDF).

**Results:**

Nineteen interviews were undertaken before reaching thematic saturation (7 nurses, 12 doctors) in emergency departments and paediatric wards from four purposively selected hospitals in Australia and New Zealand. Influencing factors were mapped to eight domains in the TDF with 21 themes identified. Main findings included: (1) Health professionals' expectations of HFNC therapy on patient deterioration, work of breathing and oxygenation; (2) Staff emotions relating to concern and anxiety about deterioration and “need to do something”; (3) Social influences from other health professionals and parents and (4) Environmental factors relating to logistics of care and patient transfer considerations. These factors, combined with the ready availability of HFNC equipment and health professionals having the required skills to administer the therapy, contributed to its initiation.

**Conclusion:**

Individual/personal and contextual/environmental factors contribute to the use of HFNC therapy for infants with bronchiolitis. It is evident these influences contribute substantially to increased use, despite evidence-based guidelines recommending a more nuanced approach to this therapy. These findings will inform a targeted implementation intervention to promote evidence-based use of HFNC therapy in infants with bronchiolitis.

## Introduction

High-flow nasal cannula (HFNC) therapy is a form of non-invasive respiratory support which delivers a blend of warmed and humidified oxygen and air into the nasal passages at a flow rate of up to 2–3 L/kg/min (1). Benefits of HFNC therapy are believed to be a reduction in the work of breathing, decreased airway resistance, improved oxygenation and infant comfort therefore reducing the need for invasive ventilation (2, 3). Mechanisms include clearance of dead space of the upper airways, washout of carbon dioxide and generation of positive end expiratory pressure (PEEP) to reduce work of breathing (4–7). HFNC therapy appears safe and is associated with few complications (8–11). International consensus recommends consideration of high-flow for infants with impending respiratory failure or severe disease (8, 12).

Within Australian and New Zealand hospitals, and internationally, there has been a significant increase in the use of HFNC therapy for respiratory support of infants with bronchiolitis without a clear rationale for this change (13). Further, quantitative data suggests that HFNC is being used in excess of these recommendations (13) and potentially used inappropriately. The reason why HFNC therapy use has increased significantly is unknown, particularly when high level evidence to support the practice is lacking with no demonstrated improvement in patient focused outcomes such as hospital length of stay (9).

The Theoretical Domains Framework (TDF) is widely used for implementation research (14, 15) having been developed specifically for identifying factors influencing the behaviour of health professionals. This paper describes use of the TDF to identify factors influencing health professionals' use of HFNC therapy for infants with bronchiolitis in emergency departments (ED) and general paediatric wards in Australia and New Zealand.

## Background

Bronchiolitis is the most common respiratory condition affecting infants less than one year of age (8, 16–18). Bronchiolitis signs and symptoms are typically coryza, fast breathing, audible wheeze, cough and use of accessory muscles to breathe, resulting in respiratory distress, difficulty feeding and reduced oxygen levels. International evidence-based guidelines consistently recommend supportive care (respiratory support, supplemental hydration requiring both medical and nursing involvement) for infants with bronchiolitis (8, 12, 19, 20). In 2017 the Australia and New Zealand Paediatric Research in Emergency Departments International Collaborative (PREDICT research network) (21) developed the first Australian and New Zealand Bronchiolitis Guideline (11, 12). Following publication of key HFNC evidence, a revision of the guideline was made that recommended HFNC therapy should be used: a) in hypoxic infants (i.e., oxygen saturations less than 92%) as a rescue therapy when standard sub-nasal oxygen has failed; and b) in non-hypoxic infants its use should be limited to the Randomised Control Trial setting only (5, 11).

When used to manage bronchiolitis, HFNC therapy is delivered into the nose *via* cannula at a rate of up to 2 L/kg/min, providing support throughout the patient's respiratory cycle. HFNC therapy is proposed to act *via* a number of mechanisms [clearance of dead space of the upper airways, washout of carbon dioxide and generation of PEEP to reduce work of breathing (4–7)], although the exact individual contribution of these remains unknown. In the paediatric setting its use was initially confined to Intensive Care Units (ICUs). Over the past 10 years, HFNC therapy use is routinely seen in EDs and general paediatric wards. The decision to commence HFNC therapy is usually made by doctors and/or nurse practitioners, with decision-making potentially being influenced by registered nurses caring for the patient.

The largest randomised controlled trial (RCT) in the field demonstrated that use of HFNC therapy for infants with bronchiolitis resulted in less escalation of respiratory support, but did not result in shorter duration of oxygen therapy, lower rates of ICU admission, nor shorter hospital length of stay and suggested that only 22%–30% of infants should be considered for rescue HFNC therapy (9). A multi-centre retrospective analysis of 11,730 infants with bronchiolitis presenting to 26 hospitals in Australia and New Zealand, found HFNC therapy use was significantly higher with 53% of infants who received oxygen managed with HFNC therapy with the frequency of HFNC therapy increasing over time (13). Reasons why HFNC therapy is used at such high rates, and potentially used inappropriately, is unknown.

The TDF consists of 14 theoretical domains or grouped theories synthesised from 33 behaviour change theories and 128 theoretical constructs. The validated framework has been utilised for a range of studies exploring barriers and facilitators to evidence-based care in a range of settings, including bronchiolitis management (22–24). Using the TDF allows a theory-informed approach to understand factors, both individual/personal and contextual/environmental, that influence current use of HFNC therapy. Understanding factors influencing the uptake of evidence-based practice in the paediatric acute care environment is vital to inform future implementation strategies to promote the appropriate evidence-based use of HFNC therapy in bronchiolitis (15).

## Method

### Aim

The aim of the study was to explore the perceived factors influencing the use of HFNC therapy for infants with bronchiolitis within ED and general paediatric wards in Australia and New Zealand.

### Design

This study used a qualitative descriptive design with in-depth, semi-structured interviews. The interview schedule was developed to explore each of the TDF domains (Supplementary Material). The COnsolidated criteria for REporting Qualitative research checklist for interviews and focus groups was followed for both conduct and reporting of the study (25).

### Setting

Site selection was purposive to ensure representation across a range of paediatric health care settings; two tertiary level paediatric hospitals (one Australian and one New Zealand site) and two mixed adult and paediatric hospitals (one Australian and one New Zealand regional site) were selected. To ensure adequate paediatric presentations, each hospital was required to have a minimum total ED annual census of 20,000 patients of which at least 5,000 were children and have access to HFNC in the ED.

### Participants

Health professionals employed in either the ED or a general paediatric ward as a doctor or nurse at the study hospitals i.e., junior doctors (training in paediatrics or emergency medicine) or specialists (paediatrician or emergency specialist) and registered nurses, were invited to participate. Participants were required to have paediatric experience, including managing infants with bronchiolitis and access to HFNC therapy in their department. An invitation was forwarded to the Clinical Director and Nurse Manager to share with staff of all grades. A minimum of three participants from each site were interviewed, with at least one being a doctor and one a nurse with variation in clinical experience. All interviews were conducted in English. Nurses and doctors were excluded from participating if not currently engaged in clinical practice, enrolled nurses, medical/nursing students, nursing agency staff, or casual staff.

Recruitment was *via* email invitation sent to Clinical Directors and Nurse Managers, who forwarded the explanatory statement on to clinical nursing and medical staff. Clinical staff registered their interest directly with the study investigators, providing implied consent to participate by emailing their contact details. Participants were screened prior to commencement of the interview to ensure they met the inclusion criteria and verbal consent reconfirmed as part of the interview.

We anticipated data saturation was likely to occur within the first 12 interviews (26). We therefore planned to interview up to 20 participants to ensure multiple perspectives and sources of data were obtained across the four hospitals. When there was no emergence of new information after three consecutive interviews, recruitment was stopped after 19 interviews (27).

### Data collection

Semi-structured interviews were conducted utilising an interview guide which had been piloted and refined to ensure consistency of data collection. Each interview consisted of two parts; first exploring current bronchiolitis management and use of HFNC and second, exploring TDF domains to understand influencing factors. Follow up questions and prompts were used to allow more in-depth exploration where required. Each interview was conducted by two of three researchers. The three interviewers included a paediatric emergency nurse and a paediatric emergency nurse practitioner, both PhD candidates with extensive experience in managing infants with bronchiolitis, as well as a PhD qualified paediatric nurse researcher with a paediatric critical care background. The second interviewer was present to take field notes and ensure consistency and effectiveness of the iterative nature of the interview. Interviews were conducted face-to-face or *via* video conferencing. Interviews were recorded digitally and transcribed verbatim. Once transcriptions were checked for accuracy, the original recordings were destroyed. To ensure anonymity, participant identifiers were removed, and codes used.

### Ethical considerations

The study was approved by the Child and Adolescent Health Human Research Ethics Committee (HREC) (EC00268), Perth Children's Hospital Western Australia, with reciprocal approval at study hospitals and by Curtin University HREC. All participants were provided with information about the study and had absolute discretion to participate in the study. Participant identity and information obtained from the interview transcripts was confidential.

### Data analysis and rigor

The de-identified transcripts were imported into NVivo 10 software (28) and analysed deductively by three researchers. Utilising conventional content analysis described by Hsieh (29), data were coded to the relevant TDF domain not cross indexed. The steps followed included familiarisation of content by repeated reading of the interview transcripts, identification of key words and/or content in the responses and allocation to the relevant TDF domain. Researchers focused on the content and meaning of the text to understand decision making in use of HFNC therapy. Descriptive accounts of the similarities and differences across each of the interviews were then clustered under the coding headings to reveal the content patterns and enable development of themes.

All stages of data analysis were independently conducted by two researchers (SO and FJG). Coding and theme development were discussed by the researchers and where there were discrepancies, a third researcher (LH) was utilised to reach consensus. Review by more than one researcher minimised any individual's subjective analysis and ensured a variety of perspectives with varying expertise were incorporated. Notations recorded by the researchers provided explanation as to decision making regarding themes and coding, such as consensus agreements and team member checking to ensure confirmability of the interpretations. To ensure trustworthiness of the coding and reduce any potential bias in data interpretation, a mini-analysis was performed after the first five interviews were analysed. Dependability was addressed by researchers maintaining clearly documented notes in a logical order. An audit trail of all decisions was kept providing an auditable and credible record of the analysis process.

## Results

A total of 19 health professional interviews (12 doctors and seven nurses) were completed over a six-month period (September 2020 to February 2021). Duration of interviews ranged from 18 to 41 (median 24) minutes. Participant characteristics are presented in Table 1. Ten (53%) health professionals were working in an ED (five in regional hospitals and five in paediatric tertiary hospitals) and 9 (47%) were working in general paediatric wards (four in regional hospitals and five in paediatric tertiary hospitals). All had a minimum of two years paediatric experience and 15 (79%) had been working in their current department for two years or more at the time of interview.

**Table 1:** Participants’ characteristics.

*N* = 19	Country		Hospital type		Total
	Australia	NZ^a^	Tertiary	Secondary	
**Female (%)**	5 (38)	8 (62)	7 (54)	6 (46)	13 (68)
**Role (%)**					
Junior Doctor	0 (0)	4 (100)	2 (50)	2 (50)	4 (21)
Senior Doctor	3 (43)	4 (57)	3 (43)	4 (57)	7 (36)
Nurse	5 (62)	3 (38)	5 (62)	3 (38)	8 (42)
**Work environment (%)**					
Emergency Department	5 (50)	5 (50)	5 (50)	5 (50)	10 (52)
Paediatric ward	3 (33)	6 (67)	4 (44)	5 (56)	9 (47)
**Professional experience**					
Paediatric experience, years (range)	8.5 (2–16)	7 (2–21)	6.5 (2–14)	11 (2–21)	7 (2–21)
Time in current department, years (range)	6.5 (0.2–13)	3 (0.3–7)	3 (0.3–13)	3 (0.3–11)	5 (0.3–13)

^a^ NZ, New Zealand.

**Figure 1:**
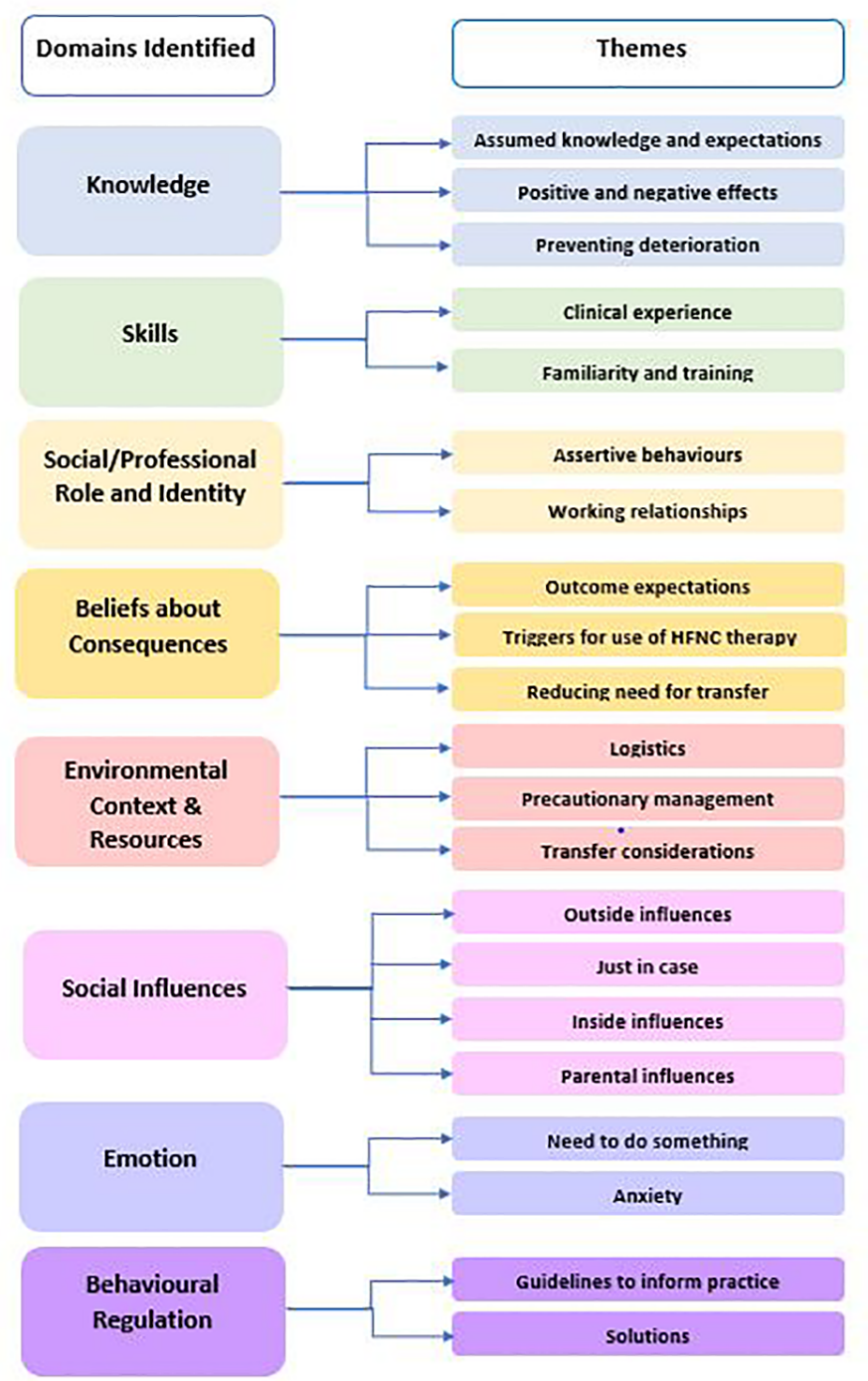
Theoretical domains framework domains and themes identified.

The key factors influencing health professionals' decision making for use of HFNC therapy were mapped to eight of the 14 TDF domains: Knowledge, Skills, Social / Professional Role and Identity, Beliefs about Consequences, Environmental Context and Resources, Social Influences, Emotion, and Behavioural Regulation with 21 themes identified (Figure 1).

### **Knowledge** (themes are shown in bolded text)

The Knowledge domain encompasses procedural knowledge or familiarity of a process or equipment usage, in addition to understanding of scientific rationale and background of the illness (14). Three key knowledge themes from participants' perspective were identified; their **Assumed knowledge and expectations** of HFNC therapy's physiological mechanisms of action, **Positive and negative effects** of HFNC therapy, and **Preventing deterioration** of the patient's condition.

Both nurses and doctors expressed a high level of **Assumed knowledge and expectations** of the physiological mechanisms of action of HFNC therapy by articulating their expected patient outcomes such as; keeping the airways open, provision of positive pressure and improving patients' oxygen levels. Participants illustrated this by describing both **Positive and negative effects** of the application of HFNC therapy. Positive effects included that the infant was more settled, appeared to clinically improve and had decreased work of breathing. There was also acknowledgment that their knowledge gained was from prior experiences, such as HFNC therapy being tolerated by some infants but not by others.

*The pros of high-flow [HFNC therapy] are that it provides a bit of positive pressure. Not as much as CPAP [Continuous Positive Airway Pressure] but not nothing compared to low-flow [oxygen]. For me, that's probably the main pro.* (Participant (*P*)1, Doctor (Dr), Ward (W))

*The high-flow [HFNC therapy] has a bit of PEEP, which is going to help keep the airways open and decrease the work of breathing… but also improve the oxygenation.* (P2, Dr, ED)

*Sometimes they'll settle really well with high-flow [HFNC therapy] because they'll feel better. Also, they could be really irritated with the high-flow [HFNC therapy]. [*P3, Nurse (Nur), W*])*

Further, their knowledge and expectations of HFNC therapy use were expressed as being that HFNC therapy will assist the infant's breathing and allow the infant to settle (decrease work of breathing), thus **Preventing deterioration.**

*If they are not on it [HFNC therapy] when they come up [to the ward], it will be commenced if they're deteriorating which would be they're either starting to work too hard and not maintaining their [oxygen] saturations, or if we think they're actually starting to work less hard and fatigue.* (P4, Nur, W)

*…my aim for high-flow [HFNC therapy] will be preventing further deterioration.* (P5, Dr, ED)

One participant explained how their knowledge of the benefits of HFNC therapy had changed.

*When I first started using it [HFNC therapy], when I first learnt about it, I thought it was only administered for work of breathing to assist with increased pressure maintaining some end-expiratory alveolar pressure to maintain airway patency and that children's work of breathing would be less with the administration of high-flow. Then, after working at a “tertiary hospital” it seemed that a main indication was mainly for hypoxemia, so to improve their oxygenation rather than their work of breathing and physiological factors affecting that. I still think it probably does both, improves oxygen levels, and assists in moderate to severe work of breathing.* (P6, Dr, W)

### Skills

The Skills domain focusses on ability and competency of skills which are often acquired through practice (14). Two key themes were identified as influencing decision making related to use of HFNC therapy: **Clinical experience** and **Familiarity and training** to set up and use HFNC therapy.

**Clinical experience** was reported by experienced nurses who:

…*have faith in their own clinical opinion,* (P7, Nur, W)

and were proactive, often suggesting to medical colleagues to initiate HFNC therapy or strongly influencing the decision to commence or not commence HFNC therapy.

* … During the wintertime, we actually have two paediatric nurses that usually are on a shift that will help with it. They're often quite forward in just starting the oxygen and ordering the equipment [HFNC therapy equipment] just straight when they bring the kid in from triage.* (P2, Dr, ED)

One senior doctor stated how skills can influence decision making to use HFNC therapy, and inappropriate use may be a result of reduced clinical skills.

*If you're very skilled at bronchiolitis, you might use it appropriately, and if you're very unskilled, you might use it because you're not sure and therefore it is “Let's just do it so it's done”.* (P8, Dr, ED)

Nurses' **Familiarity and training** to set up and use HFNC therapy equipment was identified as a facilitator for the commencement of HFNC therapy.

*The sicker ones are probably more likely to get on the high-flow [HFNC therapy] earlier if there is an experienced nurse there.* (P2, Dr, ED)

In contrast, it was reported that lack of nurses skilled in setting up and managing HFNC therapy could be a barrier, potentially resulting in delays if nurses were not comfortable or confident with HFNC therapy delivery systems.

*There might be a delay depending on nursing staffing numbers and seniority and familiarisation with equipment.* (P9, Nur, ED)

Participants were aware that specific skills were required and recognised that staff possessing those skills were essential to be able to deliver HFNC therapy. Concern was expressed regarding potential variation that existed in skill level and how this impacted on patient management.

*As long as you can troubleshoot the machine, comfortably, it's okay but I think that sometimes is a barrier for some because some people will say, “I don't feel comfortable with the machine”.* (P2, Dr, ED)

Nurses were the health professionals who were responsible for sourcing and setup of the HFNC therapy equipment, with

* … most learning pretty early on in our careers here because it's so common for us, to use high-flow.* (P3, Nur, W)

*High-flow is easy for me because I've done it for so many years now. (*P4, Nur, W)

### Social/professional role and identity

The Social/Professional Role and Identity domain incorporates the personal qualities and behaviours of the medical and nursing teams in the ward or ED settings (14). Two key themes were identified; nurses' **Assertive behaviours** and **Working relationships** with doctors.

A prominent influencing factor was the **Assertive behaviours** of nursing staff and their role and level of comfort to contact and discuss patient escalation of care with doctors.

*From a nursing point of view, I suppose we would feel comfortable trying to escalate care if required.* (P7, Nur, W)

*I'll say now we challenge doctors a little bit with decisions. We might make tactful suggestions I suppose.* (P11, Nur, W)

Nurses discussed their assertiveness, highlighting they were willing and confident to contact doctors to initiate a patient review, to provide their clinical opinion and make suggestions for changes to care. Nurses also reported variations in practice depending on doctors' responses or reactions to the assertive behaviour when discussing initiating HFNC therapy.

*It can depend on which consultant is on and what their experience has been.* (P19, Dr, W)

Further, participants described that if they were confident in their clinical knowledge and felt they had effective nursing – medical **Working relationships**, their opinions were generally respected and trusted. Medical participants also reported that they respected experienced nursing staff and encouraged junior medical staff to listen and heed advice from the nursing team.

*I do feel like the doctors that I have a good relationship with, would listen to my clinical opinion and might perhaps be swayed (…) At the end of the day, it's their call.* (P7, Nur, W)

*We strongly encourage our junior doctors to respect and listen to the voice of experienced paediatric nurses because that's where the knowledge lies.* (P12, Dr, W)

Previous positive experiences, good rapport and familiarity of the nursing staff appeared to influence the medical team and increase their trust in the nurses' clinical judgement, to heed the advice given by nursing staff rather than question it.

*Doctors that know you and know that you know what you're doing will trust you. They're like, “Yes, if you think that's what we need to do, I'm happy for you to do that”.* (P4, Nur, W)

### Beliefs about consequences

The Beliefs about Consequences domain encompasses health professionals' perception of the severity of illness and the vulnerability of the infants, and perceived benefits of the care they receive (14). Three key themes were identified; Improved **Outcome expectations**, **Triggers for the use of HFNC therapy** and **Reducing need for transfer**. Despite the lack of evidence supporting these beliefs, almost all participants spoke of **Outcome expectations** or perceived positive consequences of using HFNC therapy. **Outcome expectations** meant that HFNC therapy was regarded as an intervention or step to avoid further clinical deterioration and escalation of care. **Expected outcomes** of reduced work of breathing, supporting tired fatigued infants and quicker recovery were common influences for the commencement of HFNC therapy.

*It [HFNC therapy] lets them rest if they're fatiguing and working too hard. It [HFNC therapy] lets them just rest and recover until they're better*. (P4, Nur, W)

*I think usually with our patients they recover quicker if they're on high-flow [HFNC therapy] than if they're on regular oxygen.* (P3, Nur, W)

*High-flow [HFNC therapy] gets used more because the perception is that it works better to decrease work of breathing and improve oxygenation.* (P2, Dr, ED)

**Triggers for using HFNC therapy** included the presence of low oxygen saturations and/or patients' increased work of breathing. Doctors and nurses reported that HFNC therapy was a “*step between*” escalating to non-invasive ventilation or potentially **Reducing need for transfer** to another hospital.

*I think we've just really appreciated all of us having that step between CPAP [*non-invasive ventilation] *and low-flow [oxygen therapy] instead of having to send our children, in particular, if you don't work somewhere with an ICU on site.* (P1, Dr, W)

*It feels like we put far less children on CPAP [*non-invasive ventilation] *because we start high flow [HFNC therapy] to help them with their work of breathing. It [HFNC therapy] causes an improvement in their work of breathing. They cope with the illness much better and don't as often need to progress to CPAP [*non-invasive ventilation]*. (*P13, Dr, W)

### Environmental context and resources

The Environmental Context and Resources domain covers key constructs of equipment, materials and staffing availability and access, in addition to any environmental stressors or interactions that may be in play in any given situation (14). Three themes were identified; time of day impacted on the **Logistics** of care and staffing capabilities, reduced nursing numbers and shift times (after midnight) influenced the decision to commence HFNC therapy as **Precautionary management**, plus **Transfer considerations.**

*I’m more likely to err on the side of caution and be conservative overnight than I am in the daytime*. (P14, Dr, ED)

*Every ED is busy, and beds are at a premium, so they want kids, and I suppose adults as well, they want them either admitted or discharged as quick as possible.* (P11, Nur, W)

*It's quite often that there isn't someone who's got experience with paediatrics there (referring to a mixed ED). I think trying to set up a high-flow [HFNC therapy] system might prove difficult [in the ED]* (P15, Dr, ED)

The **Logistics** of care in relation to time of day, staffing and ability to **Transfer** a patient from ED to an inpatient ward [or Paediatric Intensive Care Unit (PICU)] was identified as influencing decision making to commence HFNC therapy:

*If it was getting towards midnight, you've got a kid who's borderline high-flow or not. You can imagine the hospital clinical manager, the ward reg, the PICU reg, and the ED, people were struggling to make a decision and the kid's hit six hours in ED. If it would help make a decision one way or another, I would absolutely be influenced by that. If PICU said, “Okay, put him on high-flow [HFNC therapy] and we'll take him.” I'd say, “Do it,” or whatever, but time of day and length of stay for sure.* (P8, Dr, ED)

*The ward won't take a child if they are acutely unwell, they have to be fairly stable, so I'd heard a rumour that sometimes they'll put them on high-flow [HFNC therapy] to stabilise, if you can call it, their work of breathing to be able to admit them to the ward.* (P11, Nur, W)

**Logistics** of care and **Transfer considerations** specific to an ED appeared to influence health professional's decision making in relation to patient admission vs. patient discharge or interhospital patient transfer. The potential consequences of decisions such as commencement of HFNC therapy, for these patients was described in the following:

*Normally, we wouldn't transfer them out [from the tertiary to a secondary hospital] if they're on high-flow [HFNC therapy] because we consider them to still be quite unwell based on if they're on high-flow [HFNC therapy].* (P4, Nur, W)

*We wouldn't transfer them out if they were on high-flow [HFNC therapy] because they wouldn't get that elsewhere.* (P2, Dr, ED)

Equipment availability was expressed by one participant as an issue:

*I think that high-flow [HFNC therapy] [equipment] potentially, logistically is slightly harder to source at times. It does take a bit more effort and logistical planning to arrange a high-flow, [HFNC therapy] not so much in ED, but often on the wards, it's a bit harder to source rather than low-flow oxygen with nasal prongs from the wall. I think that is one element that could influence staff's preference for one or the other* (P6, Dr, W)

In contrast, many participants reported that HFNC therapy equipment was *“readily available”, “well-resourced consumables”* and therefore, *“should be utilised”.* Costs of equipment and consumables, in addition to staff time was not of concern, as was included and available within hospital budgets.

### Social influences

The Social Influences domain encompasses changes in behaviour, emotion or thinking which are caused by other individuals, and may be attributed to social pressure, norms or group conformity (22). Four key themes were identified; Outside influences, Just in case, Inside influences and Parental influences.

**Outside influences** included social and interdepartmental influences such as trialling HFNC therapy as a pre-requisite for a patient to be admitted to the PICU were discussed.

*If you are getting PICU review, then often they would say, “Try high-flow [HFNC therapy] before and they would accept the child to ICU”.* (P16, Dr, ED)

Directives and advice from specialised medical teams appeared to be an influencing factor for commencing HFNC therapy.

*I think it's actually more likely to start during the day with more doctors around and for asking for more specialists around. A lot of the time, we're guided by our respiratory specialist colleagues. That's obviously an eight-to-five thing. My experience has been more [use of HFNC therapy] in the daytime actually than after-hours.* (P17, Nur, ED)

A nursing request for medical review of an infant with bronchiolitis, may prompt the initiation of HFNC therapy, due to a shared concern about patient deterioration, or as a “**Just in case**” response

*We find sometimes children have been started on high-flow [HFNC therapy] almost the second they've come in the door in ED and you come down to assess them and you think, “Oh goodness, this child looks so very well. Did they really need high-flow [HFNC therapy]?”* (P13, Dr, W)

A shared concern about clinical deterioration by nursing and medical staff from other departments (**Outside influences**), led to increased pressure on the treating medical staff to escalate treatment. In particular, nursing influence to commence HFNC therapy appeared to be very strong. Nurses were proactive to commence HFNC therapy because of their concerns about the consequences of patients tiring, working hard to breathe as well as their desire to prevent patient deterioration.

*When I'm coordinating, I sometimes go “Do you think this child would benefit from high-flow [HFNC therapy] because it is readily available, and I think they'll do really well on it?” It gets that question out there and then people go, “Actually, yes, why don't we try high-flow”[HFNC therapy]. It's very easy to use and it works wonders.* (P2, Dr, ED)

*We often get pressured into trying high-flow [HFNC therapy] when a child looks unwell, they're working hard and often too, sometime nursing colleagues and sometimes other colleagues from different department, like PICU.* (P16, Dr, ED)

Doctors also reported how they felt “*forced*” to implement HFNC therapy and alluded that a driver to commence HFNC therapy predominantly originated from the nurses they worked with (**Inside influences**). Some doctors expressed feeling pressure from senior, experienced and/or “more confident” nursing staff on the paediatric wards.

*The nurses on the children's unit will often be pushing us to escalate treatment (…) It tends to be from the more senior nurses than the junior ones. (P18*, Dr, W)

*At times, it's something that I'm forced into doing, not because I think it might make a difference* (P16, Dr, ED)

Further, initial resistance from treating doctors was not a deterrent to nurses strongly advocating for the commencement HFNC therapy.

*I've had some resistance from medical staff to try high-flow [HFNC therapy] because [oxygen] saturations are normal or above 92[%] and so why would we, but you've obviously got a very tiring baby who's got significant increased work of breathing and high resps [respiratory rate].* (P10, Nur, ED)

*It also depends on how hard the nursing staff are trying to push.* (P7, Nur, W)

Seniority and experience of medical staff was identified as a factor **(Inside influences)** either positively or negatively influencing HFNC therapy commencement. Doctors' ability to provide reassurance to nursing staff and be confident in their bronchiolitis management knowledge was identified as either a strength or a weakness of the doctor and could influence HFNC therapy commencement.

*I think when there's less experienced medical staff on, there'll probably be pressure from the nurses to escalate treatment. Whereas I think they're quite happy if it's one of the more senior people in the team who says, I assessed them. It's fine. This is why we're not doing it. There's usually some reassurance.* (P18, Dr, W)

Commencement of HFNC therapy as a result of **Inside influences** was further rationalised with knowledge about the safety of the HFNC therapy when requested by either staff or family members.

*If I've got a family member, or usually, a staff member who's convinced that this kid needs high-flow [HFNC therapy] and I know the studies show that it's safe, I'll say, Great, let's do it!* (P8, Dr, ED)

**Parental influence** appeared to play only a minor role in influencing management. Parent opinions for and against HFNC therapy appeared to be related to their own previous experience.

If associated with perceived positive outcomes, previous parental experience of HFNC therapy could potentially provide a sense of confidence in HFNC therapy:

*No, he definitely needs this and that's what he had. She [the parent] was very strict on what he'd received last time because it had worked wonders.* (P2, Dr, ED)

Some nurses reported negative opinions about HFNC therapy expressed by parents, in particular the restrictive nature of the HFNC therapy delivery system tubing. While the delivery system did not prevent the infant from being comforted, it did make this more difficult and also restricted the infant to the cot space.

*There are always families that don't want the high-flow on because then the baby is restricted to the bed and they can't move around* (P4, Nur, W)

Despite the opportunity parents had to voice concerns or express their preferences, there was an absence of parent influence on commencement of therapy.

*I don't think I've ever experienced families that have a preference for high-flow* vs. *low-flow oxygen.* (P6, Dr, W)

### Emotion

The Emotion domain is a complex reaction involving behavioural and physiological elements by which the individual attempts to deal with a significant situation or event (14). Two key themes were identified; a **Need to do something** and **Anxiety**. The most prevalent, expressed by both medical and nursing staff was a **Need to do something** to support infants with bronchiolitis. Medical and nursing participants consistently described how they “struggled” with the idea of the infant “working hard to breathe” which often prompted HFNC therapy being commenced because “we had then done something” to assist the infant to get better.

*I struggle with the idea of a child working really hard with your breathing, just managing to maintain the [oxygen] saturations with that and not being able to start flow [HFNC therapy].* (P13, Dr, W)

*I think it's the “just do something to help this kid”, (…) to be seen to be doing something as an intervention (…) It's the, “I've put something on, then that's going to make them better”, rather than the “keep calm, be supportive, do nothing”.* (P12, Dr, W)

The second theme identified was **Anxiety.** Doctors reported experiencing anxiety and discomfort when repeatedly told by a parent or nurse that something should be done for the infant. Nurses' anxiety resulted from their perceived need to repeatedly request or strongly suggest HFNC therapy commencement. Doctors' knowledge that HFNC therapy will not “*do anything*” did not deter them from commencing HFNC therapy. The emotion of **Anxiety** appeared to override underlying knowledge and rationale, as illustrated by the following:

*I think one, like a lot of things in paediatric emergency, we do it to treat ourselves. If there's a box to be ticked, let's tick it, and we get that little dopamine hit because we've done it.* (P8, Dr, ED)

*Sometimes that anxiety, when you are being told six or seven times, oh, we should be doing something, we should be doing something (…) and that product [HFNC therapy] doesn't actually do anything, but it feels like you've done something.* (P19, Dr, W)

### Behavioural regulation

The Behavioural Regulation domain covers anything aimed at managing or changing objectively observed or measured actions of a person (22). Two key themes were identified; **Guidelines to inform practice** and **Solutions.** Clinical guidelines and algorithms of care are common and useful resources available in most EDs and ward areas. Awareness of bronchiolitis **Guidelines to inform practice** was varied. Many participants reported awareness of a guideline being available, yet adherence to the guideline recommendations were never mentioned. Most participants from secondary hospitals referred to a guideline that originated from a tertiary hospital, with others reporting availability of local or national guidelines. Despite the apparent awareness of existing guidelines, they were not referred to nor routinely used to guide practice.

*I think the guidelines for bronchiolitis and high-flow [HFNC therapy] do exist, (…) They're just not something that I refer to frequently* (P6, Dr, W)

The gap between evidence and clinical practice was recognised and **Solutions** suggested, such as a pathway/algorithm and or using a reminder or prompt. Participants believed availability of such tools would be helpful to guide bronchiolitis management.

*If there was a basic pathway (…) Just something that would flag someone to think, “Well, if you're giving them oxygen, just pause one second. Do you want to try high-flow [HFNC therapy] or high-flow plus oxygen, or just oxygen?” How sick are they, in the scheme of things?* (P2, Dr, ED)

*I think having something (like an algorithm or similar… that reminds us of when we should be thinking of high-flow[HFNC therapy], what those thresholds are to be thinking about going from low-flow [oxygen] to high-flow [HFNC therapy] would be really helpful (…) especially in the heat of the moment with an unwell child.* (P1, Dr, W)

## Discussion

Supportive management is the mainstay of treatment for infants with bronchiolitis (8, 20). While HFNC therapy has a role in the supportive management, evidence suggests its use in recent years has increased above the expected proportion of infants who require escalation to this therapy (13), not only in Australia and New Zealand but also internationally (30). In this qualitative study, our theoretical approach used the TDF to identify key factors perceived to influence clinicians' use of HFNC therapy in infants with bronchiolitis. Eight domains were identified; Knowledge, Skills, Social/Professional Role and Identity, Beliefs about Consequences, Environmental Context and Resources, Social Influences, Emotion and Behavioural Regulation. These domains are likely to be contributing factors to increased use of HFNC therapy over recent years. This is the first paper using the TDF to describe factors influencing use of HFNC therapy in infants with bronchiolitis and will contribute to understanding why there has been increased use outside of clinical guideline recommendations and provide targets for future improvement efforts.

It is understandable that the level of clinical experience and skills available may impact on the care an infant with bronchiolitis receives. In this study, participants perceived they possessed a high level of clinical experience and knowledge of HFNC therapy and the required associated skills to enable set up and delivery of oxygen *via* this modality. Their desire for a treatment or intervention to reduce infants' increased work of breathing, in combination with perceived positive outcome expectancies from HFNC therapy, strongly facilitated the commencement of HFNC therapy. Both medical and nursing participants reported consideration to use HFNC therapy for infants with increased work of breathing without evidence of hypoxaemia, clearly demonstrating a gap between clinical practice and evidence-based clinical guideline recommendations (11). Many participants indicated they were aware of clinical guidelines, however, failure to follow the guideline appeared to be driven by emotion, social and professional role and identity, social influences and beliefs about consequences.

The actual or potential benefits and the clinical evidence-base available for use of HFNC therapy does not appear to add weight to final decision making. Further, it appears HFNC therapy may, at times, be administered when not appropriate, thus exposing the infant to a therapy with no benefit and potential risk. Having the clinical skills and readily available equipment seems to have only compounded the ease for which this therapy can be initiated.

The evidence base for HFNC therapy is reasonably robust. To date, over 2,000 infants from eight RCTs have been randomised to HFNC therapy vs. standard sub-nasal oxygen in ward or ED settings (8–11, 31). These figures are comparative to the number of infants who have been enrolled in RCTs comparing epinephrine and dexamethasone to placebo in bronchiolitis (n > 2,000 infants for each comparison), for which there is high-level evidence not supporting either treatment by itself (8). Together these three treatments have the largest evidence base from which international guidelines use to make treatment recommendations for infants with bronchiolitis (8). The eight RCTs of HFNC therapy confirm that it reduces escalation of respiratory support for hypoxic infants and does not result in increased adverse events. However, HFNC therapy does not lower the rate of admission to ICU and there is no robust evidence that it shortens time on oxygen therapy or hospital length of stay. In comparison to the prominently double blinded placebo controlled RCTs for evaluations of epinephrine and dexamethasone in bronchiolitis, the RCTs of HFNC therapy have been unblinded potentially affecting the overall quality of the evidence base (8–11, 31). A further suggested benefit of HFNC therapy is an increase in infant comfort when used for bronchiolitis. This outcome has been assessed in one RCT comparing HFNC therapy with standard sub-nasal oxygen (n = 202), the authors found a one point difference on a 5-point Likert scale, the clinical significance of which is uncertain (10). Together, these data have resulted in recommendations in Australia and New Zealand to use HFNC therapy in hypoxic infants as a rescue therapy when standard sub-nasal oxygen has failed (5, 11).

Beliefs about Consequences and Social Influence were two domains within the TDF identified to have strong influence on use of HFNC therapy. Nurses have a key role in delivering supportive care to infants with bronchiolitis, therefore their influence on bronchiolitis management is unsurprisingly strong. Nurses' beliefs about positive benefits of HFNC therapy along with doctors' knowledge that despite no benefit there was a belief the therapy will not do any harm to the patient, result in the commencement of therapy. Social influencing behaviours such as questioning and making suggestions about delivery of care to medical staff also had a strong influence on practice. When nursing staff had increased concern about patients' condition, they had confidence to challenge medical staff's decision making. Medical staff respected and valued the nursing experience and knowledge, often complying with nurses' recommendations for HFNC therapy. The potential negative that HFNC may increase health care costs (9) did not appear to be an influencing factor.

A recent study used the TDF to understand factors contributing to variations in several key areas of bronchiolitis management: the use of chest x-rays, salbutamol, corticosteroids antibiotics and adrenaline (23). Their study identified the importance of collaboration, clinical relationships and nurses' confidence in their clinical knowledge as crucial in guiding junior doctors' management of bronchiolitis. In contrast to their findings that these relationships resulted in greater evidence-based care, our study found that nursing staff's confidence and assertiveness may have contributed to excessive and inappropriate use of HFNC therapy (13). As advocates for the infants, nurses in this study, reported they provided input to doctors after their assessment of the infant's condition. This is similar to findings by Chandler, where nurses were empowered when doctors collaborated with them and considered nursing input in their decision making (32). It is widely assumed that empowerment of nurses in the workplace will lead to better care and positive patient outcomes (33), however, this was not always apparent in our study findings. To promote positive patient outcomes, nursing empowerment must be accompanied by the application of sound clinical evidence and knowledge.

Emotion appeared to be a major influence for using HFNC therapy in bronchiolitis. Participants recognised that decision making to commence HFNC therapy was often driven by concern or a “need to do something”. This concern and at times, anxiety, appeared widespread. This finding in relation to the use of HFNC therapy is consistent with findings in other studies (23, 34). Jiménez-Herrera et al. (2020) described the moral emotions arising from nurses working in emergency care situations, and identified positive feelings arose when nurses felt that they had been able to respond to a patient need, and care delivered had met its objectives. Nurses felt they were the patients' advocates, and in our study, we confirmed this advocacy role. Nurses were willing to question and suggest what they believed to be more appropriate care (i.e., HFNC therapy).

Our study has identified a range of contributing factors for the increased and potentially inappropriate use of HFNC therapy in Australian and New Zealand hospitals. In recent years, the increasing need for de-implementing inappropriate health interventions has become an international focus. The Choosing Wisely (CW) campaign has rapidly spread world-wide since its introduction in 2012 and aims to reduce wasteful and unnecessary medical treatments and interventions (35). Many paediatric societies have embraced this program, publishing recommendations focussing on bronchiolitis and the medicalisation of its management (35). Reducing the use of inappropriate health interventions is important to minimize patient harm, maximize resources, and improve evidence-based health care delivery (36). Emotion was identified as a potential reason for utilising therapies of no benefit as “doing so feels safer, alleviates uncertainty and due to pressure and anxiety from families and clinicians” (35). Further barriers to complying with evidence-based guidelines ironically, are the ready availability of HFNC equipment and the belief that HFNC improves infants’ outcomes. It is apparent that the potential increase in length of hospitalization, healthcare costs and other iatrogenic harm such as discomfort and localized skin damage, are not be appreciated or considered (37).

Changing clinicians’ beliefs and practice is challenging. Addressing influencing factors with appropriate behaviour change techniques is more likely to have success in changing beliefs and practice, than choosing techniques by chance alone (36). As reported by Schmutz et al. (2013), the quality of teamwork can directly influence the quality and safety of the health care provided to a patient, making it imperative to target both doctors and nurses to improve team processes and the delivery of safe and quality care consistent with current evidence-based guidelines.

### Strengths and limitations

A strength of this study was purposive sampling from a range of hospitals where infants with bronchiolitis are managed in Australia and New Zealand. The 19 interviews reached thematic saturation across eight domains of the TDF. Although we purposively sampled for participant diversity, there was under representation of nursing staff at one site and this is a limitation of this study. In addition, the views of participants may not reflect the views of all health professionals who manage bronchiolitis. As participation was voluntary it may have resulted in a biased sample of those with extreme views regarding HRNC therapy, either positive or negative. Although this was not evident from the interviews and transferability of results is enhanced by participants being from four sites in Australia and New Zealand, representing a diverse population who use HFNC therapy for paediatric patients. The participants were comparable to those interviewed by Haskell et al. (2020) who explored factors influencing the uptake of five evidence-based bronchiolitis guideline recommendations. Similarities of findings between the two studies reinforces the likelihood that the findings are reflective of Australian and New Zealand doctors and nurses managing infants with bronchiolitis. Generalisability outside of Australia and New Zealand may be limited.

Our findings are based on medical and nursing accounts of their behaviour and management of infants with bronchiolitis and the use of HFNC therapy. Ultimately, identification of these influencing factors will allow the design of interventions using appropriate behaviour change techniques to improve the appropriate use of HFNC therapy in infants with bronchiolitis (38).

## Conclusion

Using a theoretical approach, this study has identified factors influencing use of HFNC therapy in infants with bronchiolitis, by both nurses and doctors in ED and paediatric wards in Australia and New Zealand. The TDF provided a framework to systematically assess and identify a range of factors, both individual/personal and contextual/environmental influencing decision making of nurses and doctors to use HFNC therapy. Despite having clinical knowledge and evidence-based guidelines for appropriate use of HFNC therapy, HFNC use is driven primarily by four key TDF domains of emotion, social and professional role and identity, social influences and beliefs about consequences.

Identifying and understanding influencing factors will assist with design of interventions to reduce non-evidenced-based use of HFNC therapy. Addressing social influences, emotional drivers such as anxiety, knowledge and adherence to evidence-based guidelines will be important. Targeting interventions to nurses and doctors caring for infants with bronchiolitis should be a priority.

## Data Availability

The original contributions presented in the study are included in the article/**Supplementary Material**, further inquiries can be directed to the corresponding authors.
