# Supplementary material for: Factors influencing health professionals' use of high-flow nasal cannula therapy for infants with bronchiolitis – A qualitative study
Source: Front Pediatr. 2023 Mar 16;11:1098577. doi: 10.3389/fped.2023.1098577 (PMC10060553; doi:10.3389/fped.2023.1098577)
Supplement: Supplementary file 1 [file Datasheet1.docx]

| High Flow Nasal Cannula Therapy – Factors influencing care in thepaediatric setting |
| --- |

**Interview Script - Eligibility**

Participant Eligibility will be checked prior to commencement of the interview by use of the following questions. A clinician is eligible to participate if they are a doctor or nurse and the answers to question 1, and 4 is Yes and the answer to question 3 is No.

| **Question Number** | **Screening Question** | **Response (circle)** |
| --- | --- | --- |
| 1 | Are you currently employed in either emergency department or a general paediatric ward as a medical or nursing employee? | Yes / No |
| 2 | What is your profession? | Doctor or Nurse |
| 3 | Are you employed to work at the ‘nominated’ health service by a nursing agency, locum service or as a casual staff member? | Yes / No |
| 4 | Have you been responsible for the management and care of infants with bronchiolitis and have access to HFNC therapy in your department? | Yes / No |
| **Eligibility** | | **Yes / No** |

*Turn on recorder*

**Interview Script - Consent**

Thank you for taking part in this study. As explained in the Study Information statement, this study aims to identify and explore the factors influencing the use of High Flow Nasal Cannula (HFNC) therapy for infants with bronchiolitis.

**Verbal Consent**

You have been provided with a copy of the Participant Information regarding the study. Can you please confirm that you are happy to participate in the High Flow Nasal Cannula Therapy – Factors influencing care in the paediatric setting study and proceed with this interview?

**Yes □ No □**

**Interview Script - Introduction**

The interview contains a series of questions about how infants with Bronchiolitis are managed in your hospital and what factors may influence your management. We are planning to speak with both nursing and medical staff from a range of hospitals in order to learn from their knowledge and experiences in regard to the care that is being given to these infants.

The interview should take approximately 30-45 minutes. You are free to decline any questions you would prefer not to answer and if you need to cease the interview at any time, please let me know. We can reschedule an alternative time if required.

Before I start do you have any questions you would like to ask me?

**Demographic / Background Information**

1. Gender : □ Male □ Female □ Other
2. What department/ward area do you currently work in? _____________________________
3. How many years have you been working in your current department? __________________
4. Have you used HFNC in PICU previously? _________________

**Provision of Oxygen Therapy for infants with bronchiolitis – open questions**

1. Can you explain to me how infants with bronchiolitis are managed in your department/ward?
2. What do you think are the key indicators for deciding how an infant with bronchiolitis should be managed/cared for?
3. What do you/your department/ward consider low oxygen saturations?
4. What happens when an infant with bronchiolitis in your department/ward has low oxygen saturations?
5. What happens when an infant with bronchiolitis in your department /ward has increased work of breathing but OK oxygen saturations?
6. When is HFNC therapy started in your department/ward for infants with bronchiolitis?

**Interview Script - Prompts**

***The following information is for the interviewer only and will not be read to the participant, but used as prompts during the interview if required.***

The following table groups a number of barriers and facilitators in relation to the use of oxygen therapy and the theoretical domain framework. Depending on the interview in progress and the topics being discussed, these prompts may be used to further explore domains.

| **TDF Domains**  **(Definition)** | **Prompt Questions** |
| --- | --- |
| Knowledge  *[An awareness of the existence of something]* | How would you decide whether an infant with bronchiolitis requires oxygen therapy?  How do you make these decisions or what makes you choose this care? |
| Skills  *[An ability or proficiency acquired through practice]* | What skills / experience do you think are needed to use HFNC therapy vs. standard O2 therapy and can you tell me about the pros and cons of each?  Is staff skill Mix a factor? |
| Social professional role and identity  *[A coherent set of behaviours and displayed personal qualities of an individual in a social or work setting]* | Do you think deciding which oxygen therapy to be used is part of your role?  What happens if you question why it has been prescribed (by someone else)?  Who can you discuss this with in your area?  Is there any time that you have felt pressured to use HFNC over standard therapy? Describe. |
| Beliefs about capabilities  *[Acceptance of the truth, or validity about an ability, talent or facility that a person can put to constructive use]* | Have you experienced challenges in negotiating with families / colleagues who may want HFNC therapy over standard O2 therapy? How do you manage this? |
| Optimism  *[The confidence that things happen for the best or that desired goals will be attained]* | Do you feel that giving HFNC therapy improves outcomes / or alters your decision making processes? |
| Beliefs about consequences  *[Acceptance of the truth, reality, or validity about outcomes of a behaviour in a given situation]* | Benefits / disadvantages of using HFNC therapy vs. standard O2.  What is said / not said by colleagues if you use or don’t use HFNC therapy? |
| Reinforcements  *[Increasing the probability of a response by arranging a dependent relationship, or contingency, between the response and a given stimulus]* | Incentives / disincentives to use HFNC therapy/standard O2 therapy? |
| **TDF Domains**  **(Definition)** | **Prompt Questions** |
| Intentions  *[A conscious decision to perform a behaviour or a resolve to act in a certain way]* | Do you consciously think about whether or not to give HFNC therapy in infants with bronchiolitis? |
| Goals  *[Mental representations of outcomes or end states that an individual wants to achieve]* | Would the use of HFNC ever impact on whether a child was admitted or transferred to another hospital for management? |
| Memory, attention and decision processes  *[The ability to retain information, focus selectively on aspects of the environment and choose between 2 or more alternatives]* | Is the use/application of HFNC something you do routinely/frequently? |
| Environmental context and resources  *[Any circumstances of a person’s situation or environment that discourages or encourages the development of skills and abilities, independence, social competence, and adaptive behaviour]* | What influences your decision to use HFNC therapy? Eg. Number/ skill mix of nursing staff, bed or equipment availability... time of day/weekend  Attempt to reduce LOS/transfer? |
| Social influences  *[A coherent set of behaviours and displayed personal qualities of an individual in a social or work setting]* | Do you seek opinion of colleagues before using HFNC therapy? If so, who?  What are the views of your colleagues? |
| Emotion  *[A complex reaction pattern, involving experiential, behavioural, and psychological elements, by which the individual attempts to deal with a personally significant matter or event]* | Are there times when it is difficult to know whether to give HFNC therapy or use standard oxygen therapy? Is there someone you can discuss this with?  Do staff or families opinions influence your decision to use or not use HFNC? |
| Behavioural regulation  *[Anything aimed at managing or changing objectively observed or measured actions]* | Are there any guidelines to guide your practise?  What do you think would help you / your colleagues / environment change practice in regards to use of HFNC therapy? |
